# A novel approach to mapping the atrial ganglionated plexus network by generating a distribution probability atlas

**DOI:** 10.1111/jce.13723

**Published:** 2018-10-05

**Authors:** Min‐Young Kim, Markus B. Sikkel, Ross J. Hunter, Guy A. Haywood, David R. Tomlinson, Muzahir H. Tayebjee, Rheeda L. Ali, Chris D. Cantwell, Hanney Gonna, Belinda C. Sandler, Elaine Lim, Guy Furniss, Dimitrios Panagopoulos, Gordon Begg, Gurpreet Dhillon, Nicola J. Hill, James O’Neill, Darrel P. Francis, Phang Boon Lim, Nicholas S. Peters, Nick W. F. Linton, Prapa Kanagaratnam

**Affiliations:** ^1^ Myocardial Function Section, Imperial Centre for Translational and Experimental Medicine, Imperial College London London UK; ^2^ Imperial Centre for Cardiac Engineering, Imperial College London London UK; ^3^ Department of Cardiology Imperial College Healthcare NHS Trust London UK; ^4^ Department of Cardiology The Barts Heart Centre, St Bartholomew’s Hospital, Barts Health NHS Trust London UK; ^5^ Department of Cardiology Derriford Hospital, Plymouth Hospitals NHS Trust Plymouth UK; ^6^ Department of Cardiology Leeds General Infirmary, Leeds Teaching Hospitals NHS Trust Leeds UK

**Keywords:** atrial fibrillation, autonomic nervous system, ganglionated plexus

## Abstract

**Introduction:**

The ganglionated plexuses (GPs) of the intrinsic cardiac autonomic system are implicated in arrhythmogenesis. GP localization by stimulation of the epicardial fat pads to produce atrioventricular dissociating (AVD) effects is well described. We determined the anatomical distribution of the left atrial GPs that influence atrioventricular (AV) dissociation.

**Methods and Results:**

High frequency stimulation was delivered through a Smart‐Touch catheter in the left atrium of patients undergoing atrial fibrillation (AF) ablation. Three dimensional locations of points tested throughout the entire chamber were recorded on the CARTO™ system. Impact on the AV conduction was categorized as ventricular asystole, bradycardia, or no effect. CARTO maps were exported, registered, and transformed onto a reference left atrial geometry using a custom software, enabling data from multiple patients to be overlaid. In 28 patients, 2108 locations were tested and 283 sites (13%) demonstrated (AVD‐GP) effects. There were 10 AVD‐GPs (interquartile range, 11.5) per patient. Eighty percent (226) produced asystole and 20% (57) showed bradycardia. The distribution of the two groups was very similar. Highest probability of AVD‐GPs (>20%) was identified in: inferoseptal portion (41%) and right inferior pulmonary vein base (30%) of the posterior wall, right superior pulmonary vein antrum (31%).

**Conclusion:**

It is feasible to map the entire left atrium for AVD‐GPs before AF ablation. Aggregated data from multiple patients, producing a distribution probability atlas of AVD‐GPs, identified three regions with a higher likelihood for finding AVD‐GPs and these matched the histological descriptions. This approach could be used to better characterize the autonomic network.

## 1 INTRODUCTION

The autonomic nervous system (ANS) regulates normal cardiac function but is also implicated in pathological processes such as arrhythmogenesis.^1^, ^2^, ^3^, ^4^, ^5^ Such proarrhythmic changes are likely to be mediated by the intrinsic cardiac ANS, which is a complex network of ganglionated plexuses (GPs) around the epicardium. There is also a constant interaction with the extrinsic GPs (stellate, middle and superior cervical GPs) modulating the electromechanical function of the heart as a result of signals from the spinal cord, medulla, and higher centers. These different hierarchical neural centers have afferent, efferent, and local circuit neurons that interdependently interact with each other by receiving inputs from physiological and pathological stressors.^6^

The left atrium (LA) innervation is of particular interest, as it has been proposed as a potential target for AF therapies. Anatomical studies of postmortem human hearts identified 800 GPs per heart, with the densest collection (50% of all cardiac GPs) in the hilum between the posterior and posterolateral surfaces of the LA. GP sites varied widely between the hearts but with three main common clusters (superior left, posteromedial left, and posterior right) and extending anteriorly into the interatrial septum (interatrial septal GP).^7^, ^8^ Further studies indicated the highest density of nerve fibers were in the ostium and antrum of the pulmonary veins (PV) and posterior portion of the LA.^9^, ^10^ Although the GPs are epicardial, smaller nerve fibrils penetrate the endocardium allowing for conduction of electrical stimulation from the endocardium to the epicardial GPs.^9^, ^11^, ^12^

High frequency stimulation (HFS) of canine epicardial GPs reduces heart rate and blood pressure (BP).^13^, ^14^, ^15^, ^16^, ^17^ Similarly, changes occur in patients with endocardial HFS directly under the epicardial GP.^1^, ^18^, ^19^, ^20^, ^21^, ^22^ Therefore the neural network can be accessed and influenced by stimulation of endocardial sites.^23^, ^24^ The atria will fibrillate when HFS is applied due to high rate capture, but at atrial GP sites there will also be ventricular rate slowing with a more than or equal to 50% increase in the mean RR interval compared to baseline. This response has been described as a “vagal response, vagal reflex, bradycardia, atrioventricular (AV) node block, and asystole” in other studies.^25^, ^26^, ^27^, ^28^, ^29^ For greater specificity, we will use the term “atrioventricular dissociating GP” (AVD‐GP) for GP sites that show AV dissociation.

In this study, we have examined the distribution of AVD‐GPs in the human LA with high density HFS mapping in patients having AF ablations. We then combined their LA geometries to create a probability atlas of AVD‐GPs.

## 2 MATERIALS AND METHODS

### 2.1 Patients

Twenty‐eight patients with symptomatic, paroxysmal AF undergoing first ablation procedure were recruited to the study from four centers. Patients gave written informed consent and the study had ethics approval from the Health Research Authority and the Local Research Ethics Committee. Antiarrhythmics were stopped for five half‐lives before each procedure. Patient characteristics are shown in Table 1.

**Table 1:** Demographic characteristics of patients recruited to this study

Demographic characteristics	
Age, y	64 ± 10
Sex, male/female	17/11
BMI, kg/m^2^	27.5 ± 4.8
LVEF, %	61 ± 4.5
LA diameter, mm	3.8 ± 0.6
CHA_2_DS_2_‐VASc	1.7
Stroke/TIA	2
CAD	3
HTN	12
DM	3
Fluoroscopy time, min	23.2 ± 13.9
Procedure time, min	248.9 ± 123.4
HFS mapping time, min	63.3 ± 24.8

Numbers in the right column represent mean ± SD.

Abbreviations: BMI, body mass index; CAD, coronary artery disease; DM, diabetes mellitus; HFS, high frequency stimulation; HTN, hypertension; LA, left atrium; LVEF, left ventricular ejection fraction; TIA, transient ischemic attack.

### 2.2 Mapping for AVD‐GP

Patients had general anesthesia and a transesophageal echocardiogram to rule out cardiac thrombus. A decapolar catheter was positioned in the coronary sinus. A quadripolar catheter was inserted into the right ventricle that provided clearer ventricular signals within the HFS noise. After transseptal puncture, a 20‐pole circumferential catheter (LassoNav; Biosense Webster Inc, Diamond Bar, CA) was used to create a respiratory‐gated three‐dimensional electroanatomic map of the left atrium (CARTO™; Biosense Webster, Inc). It was then placed in one of the PVs. A bipolar 3.5 mm irrigated‐tip contact force sensing ablation catheter (Smart‐Touch; Biosense Webster, Inc) was positioned on a stable endocardial surface with a target contact force more than 3 g before delivering HFS. BP was continuously monitored using a radial arterial line. Heparin was administered throughout the cases to maintain the activated clotting time more than 300 seconds.

To locate an AVD‐GP, a S88 Grass stimulator (Astro‐Med, West Warwick, RI) was used to stimulate from the distal electrode of the ablation catheter for at least four beats to ensure there was no ventricular capture. HFS was then applied (20 Hz, amplitude, 12 V; pulse duration, 10 milliseconds) for up to 10 seconds or until asystole occurred. We aimed to map the whole atrium with evenly distributed HFS points, within 6 mm distance between points. All patients had AF induced with the first application of HFS due to local myocardial capture. All data were recorded at 1000 Hz by the electrophysiology (EP) recording system (Bard EP, Lowell, MA). RR intervals were measured from the maximum peaks of the arterial BP trace, or the right ventricular electrogram. In the event of any measurement uncertainty, HFS was repeated at the tested site. Ablation was performed after the HFS mapping protocol. Retrospectively, the number of AVD‐GPs distal or proximal to the PV isolation (PVI) ablation lines were manually counted.

### 2.3 Defining an AVD‐GP

In animal experiments, GP sites were defined as causing more than or equal to 50% increase in the mean RR interval during HFS from the baseline.^1^ The mean RR interval during HFS was measured from the time between the first R during HFS and the first R after the cessation of HFS. The baseline RR interval was defined as the mean of the 10 RR intervals immediately preceding HFS.

Asystole was the most frequent response to HFS. When this occurred, HFS was stopped and RR intervals and BP recovered quickly. We have termed these sites as “asystole AVD‐GPs” or “A‐AVD‐GPs.”

Some GP sites gave a milder prolongation of RR intervals, with no asystole. These showed a stable bradycardic response throughout the HFS. As with A‐AVD‐GP, RR intervals recovered quickly after HFS stopped. We have termed these sites as “bradycardia atrioventricular dissociating ganglionated plexus” or “B‐AVD‐GPs.”

**Figure 1:**
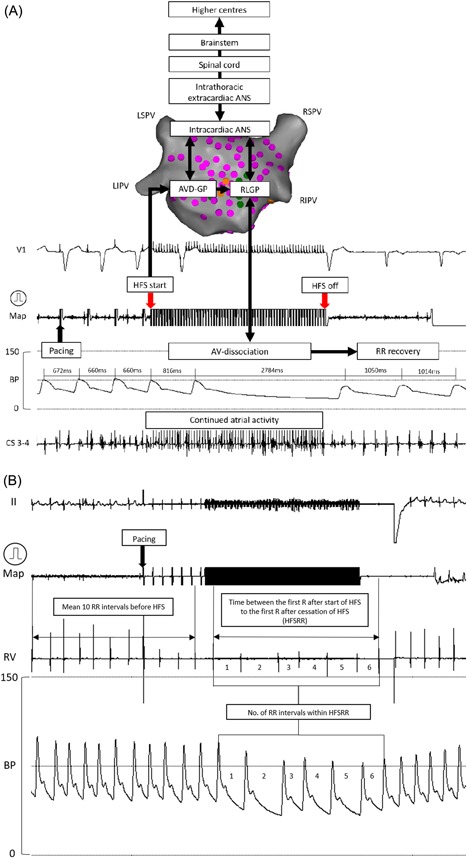
(A) Hierarchical stages of the ANS from the central to the peripheral system. First, a mapping catheter is used to pace in the endocardium of the left atrium for four beats to ensure that there is no ventricular capture. HFS is then delivered at 20 Hz, 12 V. Asystole occurs almost immediately after starting HFS. This is due to direct AV dissociation or via stimulation of the RLGP acting as the common “gateway” to the AV node. During HFS and AV dissociation, there is continued atrial activity as observed in CS 3 to 4. Due to the high voltage output, the Map electrogram only shows output signals during pacing and HFS. The RR interval recovers following cessation of HFS. This site was determined as an A‐AVD‐GP site. (B) An intracardiac recording of determination of a B‐AVD‐GP site. We have performed HFS with the same parameters as (A) but for 10 seconds. The mean of 10 RR intervals preceding HFS was 952 milliseconds. We then measured the total time duration between the first R after starting HFS and the first R after cessation of HFS (HFSRR) and averaged this to calculate the mean RR interval (1610 milliseconds). There was increase in more than 50% of the RR interval during HFS from the baseline that determined it as an AVD‐GP site. However, there was no asystole like in A‐AVD‐GP. This was therefore determined as a B‐AVD‐GP site. A‐AVD‐GP, asystole atrioventricular dissociating ganglionated plexus; ANS, autonomic nervous system; AV, atrioventricular node; AVD‐GP, atrioventricular dissociating ganglionated plexus; B‐AVD‐GP, bradycardia atrioventricular dissociating ganglionated plexus; BP, blood pressure; CS, coronary sinus; HFS, high frequency stimulation; GP, ganglionated plexus; LA, left atrium; RLGP, right lower ganglionated plexus [Color figure can be viewed at wileyonlinelibrary.com]

To distinguish the two responses objectively, we looked at the normal RR variability in our patients during AF. We randomly selected 75 samples of 20 seconds AF electrograms from our patients. For each sample, we averaged the first 10 seconds RR intervals and measured the longest RR interval in the last 10 seconds. The ratio of the latter to the mean 10 seconds RR interval was calculated. This was repeated for all 75 samples. We have performed the Shapiro‐Wilk test to confirm that the log‐transformed ratios were not significantly different to normal distribution (*P* = 0.56). This confirmed a log‐normal distribution of the ratios. In a one‐tailed log normal distribution, 2.33 standard deviations above the mean gave the ratio of 2.6 whereby less than 1% of single RR prolongation during HFS is a false‐positive AVD‐GP. Therefore, any single RR prolongation during HFS that was more than 2.5 times the average 10 seconds RR interval before HFS was defined as an A‐AVD‐GP site. This was equivalent to more than 150% increase in the single RR prolongation from the baseline (Figure 1A). Any ratio below this threshold and within the definition of an AVD‐GP (> 50% increase in the average RR interval from the baseline) was termed “B‐AVD‐GP” (Figure 1B).

**Figure 2:**
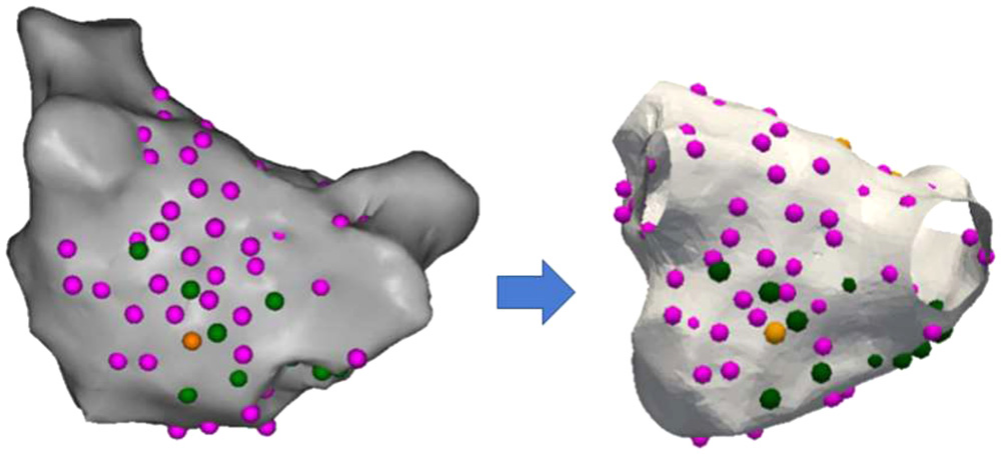
An example of a patient’s CARTO left atrial image (posterior‐anterior view) with A‐AVD‐GP (green), B‐AVD‐GP (orange), and high frequency stimulation negative sites (pink) transformed onto a reference left atrial shell. A‐AVD‐GP, asystole atrioventricular dissociating ganglionated plexus; B‐AVD‐GP, bradycardia atrioventricular dissociating ganglionated plexus [Color figure can be viewed at wileyonlinelibrary.com]

Using the CARTO Tag system, A‐AVD‐GP were marked green and B‐AVD‐GP were marked orange. Negative responses to HFS were marked pink (Figure 2).

### 2.4 Registration

LA fast anatomical map of all the patients were exported from CARTO. A representative LA anatomy was chosen as a reference shell and all patient data was coregistered onto this geometry using a semiautomated process.^30^ This shell was chosen as having a “typical” distribution of the four pulmonary veins.

Four sets of circumferential landmarks, with each pair of landmarks separated by a constant angle were automatically generated along the intersection of PV‐atrial junctions and a plane, at the point of maximum geometric curvature. Landmark registration followed by nonrigid surface registration was used to compute mapping from each patient‐specific geometry to the reference LA surface. An automated angle‐based fiducial‐point selection algorithm was used to choose the optimal landmarks on the PV‐atrial junctions for each patient, which minimized the target registration error (TRE) when coregistered to the reference shell. TRE is defined as the average distance between the true position of landmarks on the target reference shell, which were not used during the registration process, and their position when the corresponding landmark on the source shell was mapped under the computed transformation.

This enabled AVD‐GP and negative HFS points to be accurately transformed onto the reference LA shell. An example of a registration with data points transformed on to the common shell is shown in Figure 2.

### 2.5 Probability distribution atlas of AVD‐GP

A probability of 1 was applied to AVD‐GP sites and 0 for sites tested with HFS that did not evoke an AVD response. Each measurement site was weighted by a Gaussian kernel with a variance of 5 mm, which characterized the uncertainty in the measurement of the site location as a result of catheter movement. The probability that an arbitrary test point within the reference atrial surface that has an AVD response was then computed per patient. This was the sum of the product of probabilities of nearby measured sites with their respective weight kernels evaluated at the required point that was then normalized by the sum of the weight kernels evaluated at the point. Resulting maps were then averaged across the patients.

### 2.6 Statistics analysis

All continuous variables were expressed as mean and standard deviation (mean ± SD) or median and interquartile range (IQR), unless otherwise explicitly stated. Shapiro‐Wilk test was used for normality test of log‐transformed figures.

## 3 RESULTS

**Figure 3:**
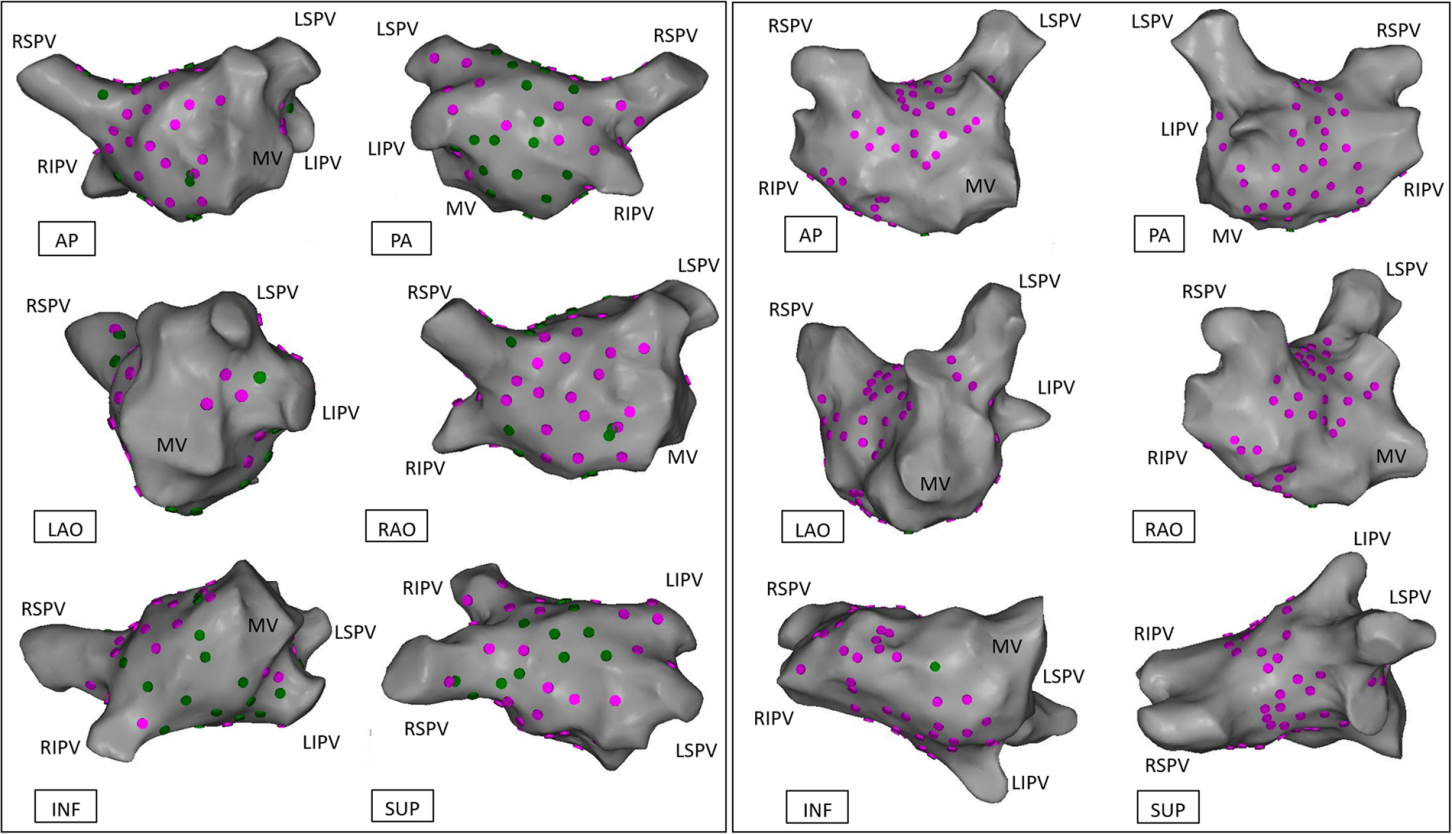
Examples of patients with the lowest and the highest number of AVD‐GPs. The left panel shows the highest number of AVD‐GPs (31) and the right panel shows the lowest number (1) of AVD‐GPs. Pink dots indicate negative HFS sites, green dots are A‐AVD‐GPs. Neither patients had B‐AVD‐GP. Clinical characteristics of the patients are as follows: Left panel, patient, 67 years old, male, BMI 28.1, good LVSF, LA size 3 cm, hypertensive, 72 points tested in total, 29 were A‐AVD‐GP; right panel, patient, 77 years old, male, BMI 25.5, good LVSF, LA size 3.9 cm, previous stroke, previous percutaneous coronary intervention, hypertensive, 71 points tested in total. A‐AVD‐GP, asystole atrioventricular dissociating ganglionated plexus; AP, anterior‐posterior; AVD‐GP, atrioventricular dissociating ganglionated plexus; B‐AVD‐GP, bradycardia atrioventricular dissociating ganglionated plexus; INF, inferior; LSPV, left superior pulmonary vein; LAO, left anterior oblique; LIPV, left inferior pulmonary vein; MV, mitral valve; PA, posterior‐anterior; PVI, pulmonary vein isolation; RAO, right anterior oblique; RSPV, right superior pulmonary vein; RIPV, right inferior pulmonary vein; RLGP, right lower ganglionated plexus; SUP, superior [Color figure can be viewed at wileyonlinelibrary.com]

**Figure 4:**
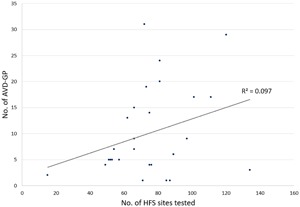
A scatter plot with 28 patients and the number of AVD‐GPs identified out of the total number of high frequency stimulation sites tested per patient. There was no clear linear relationship between the two variables. AVD‐GP, atrioventricular dissociating ganglionated plexus

We recruited 28 patients (17 males, 11 females, mean age 64 ± 10 years). Full demographics and clinical characteristics are in Table 1. The total number and the median number of HFS points tested per patient was 2108 and 74 (IQR 27), respectively. The total number and the median number of AVD‐GPs identified was 283 (13%) and 6.5 (IQR 12), respectively. The number of AVD‐GPs ranged widely from 1 to over 30 (Figure 3). There was no correlation between the number of HFS tested sites and the number of AVD‐GPs (Figure 4). Registration of all our patients to one reference shell revealed that 48% of the total area of the left atrium contained 90% of AVD‐GPs. The average TRE of our registration was 2.7 ± 1.0 mm (where “0” is perfect registration).

**Figure 5:**
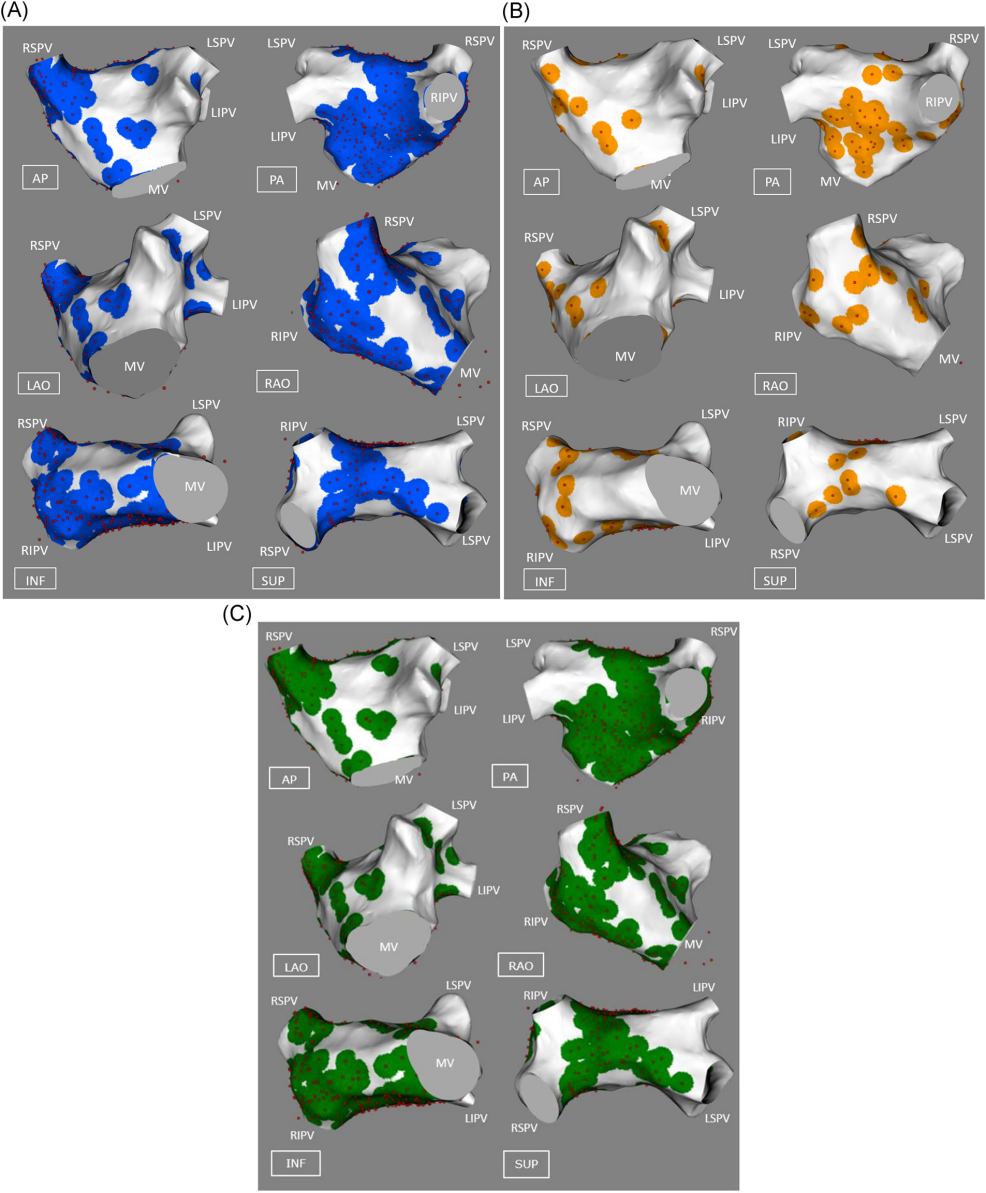
A‐AVD‐GP and B‐AVD‐GP distributions in the left atrium. (A) All the A‐AVD‐GPs identified from 28 patients on the reference left atrial shell. The red spots indicate A‐AVD‐GPs and the blue circle around each point represents the 5 mm kernel variance. (B) All the B‐AVD‐GPs identified. The red spots indicate B‐AVD‐GPs and the orange circle around each site represents the 5 mm kernel variance. Areas in white are absent of any AVD‐GPs. (C) The combination of (A) and (B) into one map of AVD‐GPs. A‐AVD‐GP, asystole atrioventricular dissociating ganglionated plexus; AVD‐GP, atrioventricular dissociating ganglionated plexus; AP, anterior‐posterior; B‐AVD‐GP, bradycardia atrioventricular dissociating ganglionated plexus; INF, inferior; LAO, left anterior oblique; LIPV, left inferior pulmonary vein; LSPV, left superior pulmonary vein; MV, mitral valve; PA, posterior‐anterior; RAO, right anterior oblique; RSPV, right superior pulmonary vein; RIPV, right inferior pulmonary vein; RLGP, right lower ganglionated plexus; SUP, superior [Color figure can be viewed at wileyonlinelibrary.com]

The total number of A‐AVD‐GP and B‐AVD‐GP was 226 (80%) and 57 (20%), respectively. The mean number per patient was 8 ± 7 (median 6; IQR 9) and 2 ± 4 (median 0, IQR 3), respectively (Table 2). The total distribution of A‐AVD‐GP and B‐AVD‐GP in the reference LA shell is shown in Figure 5A and 5B. The colors around each tested point demonstrate the degree of uncertainty (5‐mm‐radius) from the catheter movement during live cases.

**Table 2:** The breakdown of HFS points tested and AVD‐GPs identified

HFS and AVD‐GP, *n* = 28	Total, %	Mean per patient, SD	Median per patient, IQR
HFS points tested	2108	75 (24)	74 (27)
AVD‐GP identified	283 (13)	10 (8)	6.5 (12)
A‐AVD‐GP	226 (80)	8 (7)	6 (9)
B‐AVD‐GP	57 (20)	2 (4)	0 (3)

Abbreviations: A‐AVD‐GP, asystole atrioventricular dissociating ganglionated plexus; B‐AVD‐GP, bradycardia atrioventricular dissociating ganglionated plexus; HFS, high frequency stimulation; IQR, interquartile range; SD, standard deviation.

**Figure 6:**
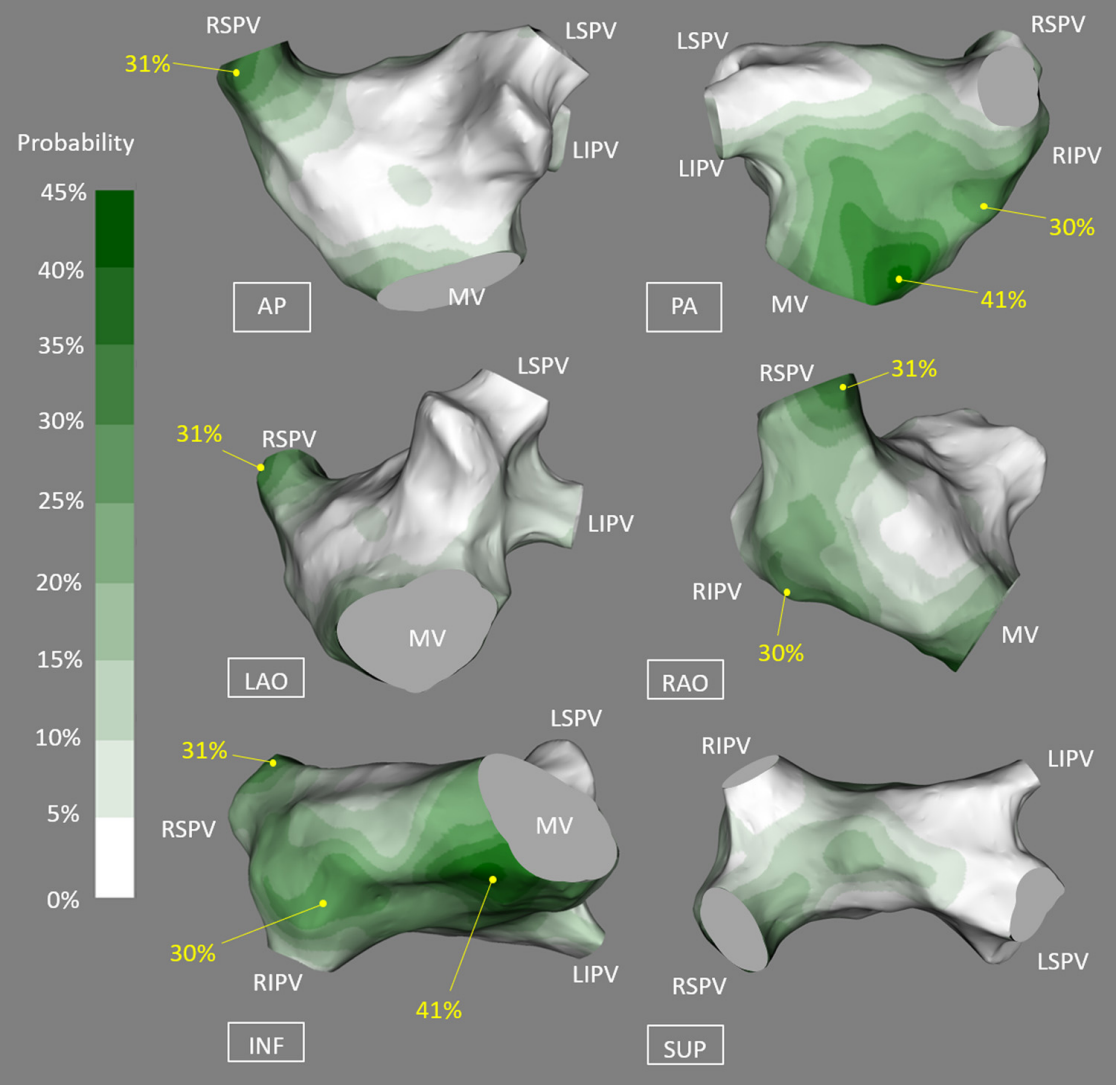
A probability distribution atlas of AVD‐GPs. The probabilities are shown in the figure from 0% to 45%, white to dark green. This resulted in clear identification of the highest probability sites of AVD‐GP to the lowest. “High probability AVD‐GP sites” were more than 20% in probability, labeled with yellow dots with corresponding probabilities. These were: inferoseptal posterior wall (41%), the RSPV antrum (31%), and the base of the RIPV in the posterior wall (30%). AP, anterior‐posterior; AVD‐GP, atrioventricular dissociating ganglionated plexus; INF, inferior; LSPV, left superior pulmonary vein; LAO, left anterior oblique; LIPV, left inferior pulmonary vein; MV, mitral valve; PA, posterior‐anterior; RAO, right anterior oblique; RSPV, right superior pulmonary vein; RIPV, right inferior pulmonary vein; RLGP, right lower ganglionated plexus; SUP, superior [Color figure can be viewed at wileyonlinelibrary.com]

The general distribution of A‐AVD‐GP and B‐AVD‐GP were in the similar regions of the LA. The combined map is shown in Figure 5C. This represents all the AVD‐GP sites from 28 patients. The largest number of AVD‐GPs were in the posterior wall of the LA and the least were in the anterior wall. By incorporating negative HFS sites to the AVD‐GP map, we have constructed a probability atlas of AVD‐GPs across the entire LA. This identified three distinct peaks with greater than 20% in probability of AVD‐GPs with a gradual decreasing gradient of probability branching outward from the peaks. These were (a) the inferoseptal aspect of the posterior wall (41%), (b) the RSPV antrum (31%), and (c) the base of the RIPV in the posterior wall (30%) (Figure 6).

**Figure 7:**
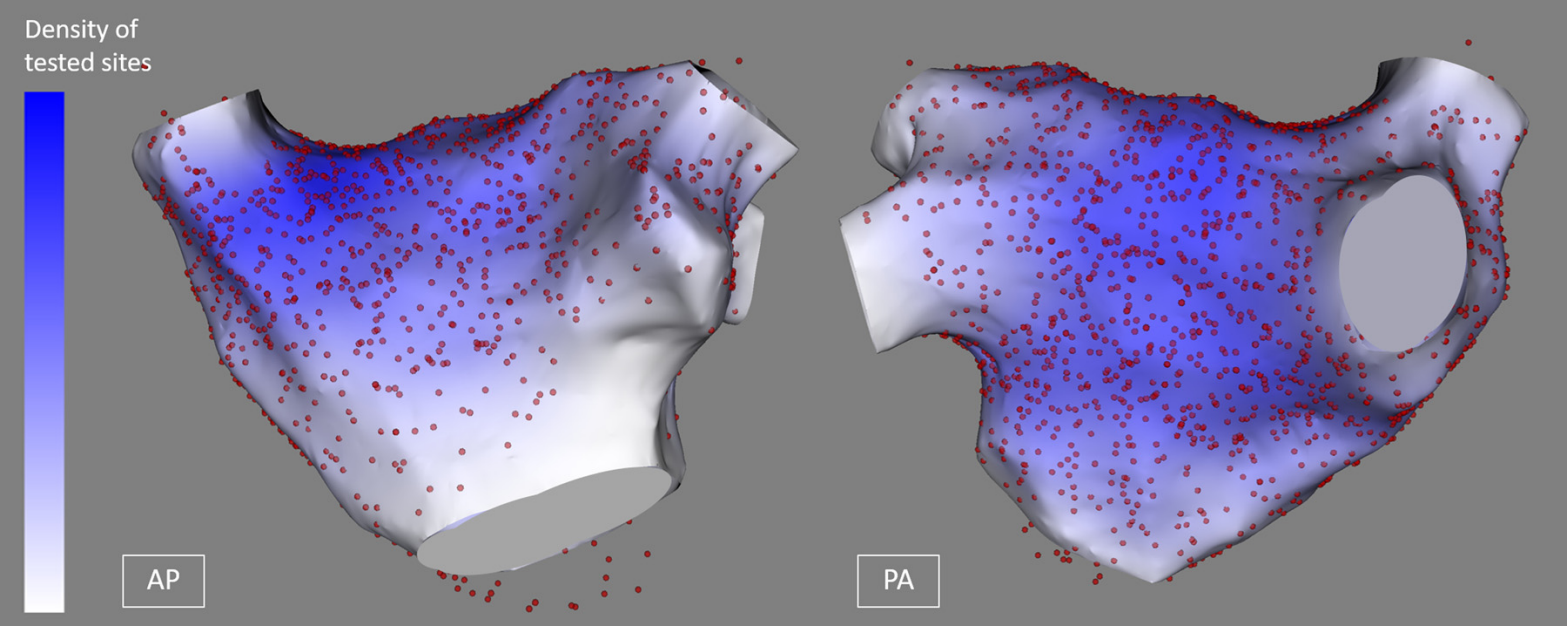
All AVD‐GPs and high frequency stimulation negative sites in 28 patients on the reference left atrial shell. Red spots represent high frequency stimulation site tested and the color scale on the left‐hand side represent the density of high frequency stimulation points tested (darkest blue representing highest density) in the left atrium. AVD‐GP, atrioventricular dissociating ganglionated plexus [Color figure can be viewed at wileyonlinelibrary.com]

The operators performed their circumferential ablation approach as per their standard practice and with the location of GP sites hidden. The location of AVD‐GP sites in relation to the circumferential ablation lines was categorized post hoc as being either included within the isolated PV antrum or being outside and unaffected by the ablation. Fifty‐three percent of AVD‐GP were outside the boundary of the PVI ablation line (Table 3). We have tested most of the left atrium (Figure 7).

**Table 3:** Locations of AVD‐GPs in 19 patients undergoing PVI

Locations of AVD‐GP in patients undergoing PVI, *n* = 19	*n* (%)
Proximal to the PVI line	
AVD‐GP	103
A‐AVD‐GP	90 (87)
B‐AVD‐GP	13 (13)
Distal to the PVI line	
AVD‐GP	118
A‐AVD‐GP	91 (77)
B‐AVD‐GP	27 (23)

The right‐hand column represents the number of AVD‐GPs and their percentages. The AVD‐GPs were manually counted retrospectively after PVI. These were categorized into “proximal to the PVI line” and “distal to the PVI line.” Ablation VISITAG™ (Biosense Webster) of CARTO were used as boundaries of PVI lines.

Abbreviations: A‐AVD‐GP, asystole atrioventricular dissociating ganglionated plexus; AVD‐GP, atrioventricular dissociating ganglionated plexus; B‐AVD‐GP, bradycardia atrioventricular dissociating ganglionated plexus, PVI, pulmonary veins isolation.

## 4 DISCUSSION

In this study we have created a probability atlas showing the regions of the LA where AVD‐GPs are most likely to be found. These areas were similar to those described in histological studies. However, the most striking finding was the marked variation in number and location of AVD‐GPs between individual patients.

GP sites are defined as having a more than or equal to 50% increase in the average RR interval from the baseline in response to HFS. However, the degree of AV dissociating effect varies with what appears to be two distinct responses: asystole and bradycardia. In our study, these were characterized separately as A‐AVD‐GP and B‐AVD‐GP. Combining all the patients’ maps, we have deduced that these two functional classes of GPs that cause AV dissociation appear to be colocated. An alternative explanation for this finding is that in fact there is only one type of AVD‐GP, with differences in response merely reflecting distance from the atrial lumen to GP, affecting the amount of current transmitted.^31^, ^32^

### 4.1 Other functional classes of GPs

GP can also be identified by delivering HFS within the refractory period of the atrium to induce atrial premature depolarization and atrial arrhythmia.^31^, ^32^, ^33^, ^34^ Our group has previously shown that these GPs identified as “ectopy‐triggering ganglionated plexus (ET‐GP)” colocate with less than half of GP sites with AV dissociating effect.^34^ We have also shown that ablation of GPs near the right upper PV influences the sinus node heart rate variability. This suggests that GPs have a spectrum of functional effects that are likely to be determined by the local neural architecture that is activated by HFS at that site. These further underlines the importance of functionally mapping the LA before considering autonomic modification.

### 4.2 High probability regions of AVD‐GP

We have identified three main regions of the LA that are abundant in AVD‐GPs, and all these sites were in close proximity to the right atrium.^7^, ^8^ The two high probability peaks in the posterior wall were located at the site where the right‐lower GP (RLGP) is expected to be. The RLGP acts as the “integration center” or “common gateway” to the AV node,^23^, ^29^ which is an important site where ablation at this site can prevent any further induction of vagal response to HFS at other GP sites.

### 4.3 GP modification in the treatment of AF

Inadvertent injury to GP sites has been assumed to occur during PVI and contribute to successes in treatment of AF.^18^ In our patients, we noted that some AVD‐GPs had been “encircled” as a part of the ablation procedure. Interestingly, more than half of AVD‐GPs remained distal to the antrally isolated myocardium.

Animal studies have shown that stimulation of GP is capable of shortening the refractory period at PVs and the atrium and ablation at these sites can abolish the effects.^22^, ^35^, ^36^ These studies led to the assumption that autonomic drive was a prerequisite for human AF. Vagal symptoms and sympathetic stressors are well‐described associations in patients with AF and have been cited as circumstantial evidence for autonomic changes being an upstream trigger in AF pathogenesis.

Although AVD‐GPs are epicardial structures, studies have shown that ablation guided by HFS mapping can eliminate the AV dissociating effects.^20^, ^21^, ^29^ This has led to a series of studies attempting autonomic modification as a therapy for AF. However, outcomes have been conflicting and has led to a class IIb classification in the 2017 HRS expert consensus for AF treatment.^37^

Even though GPs are epicardial structures, the only studies showing benefit utilized endocardial ablation.^25^, ^26^ Improved outcomes were noted in those patients who received PVI and additional ablation to “presumed GP sites” using anatomical description without any functional confirmation of GP sites.^25^ Such an approach could be considered a limited endorsement of autonomic modification, as no formal confirmation of autonomic changes was obtained. In our current study, we demonstrated that it is feasible to perform global LA mapping to determine AVD‐GP sites followed by a standard ablation procedure. Therefore, it would be possible to perform more targeted ablation procedures with formal testing rather than “blind” ablation. The study of “selective” AVD‐GP ablation tested an average of 37 sites and ablated five AVD‐GP sites.^28^ In contrast, our study needed an average of 75 sites to be tested to get sufficient coverage of the LA, identifying average 10 AVD‐GPs per patient. Therefore, studies of AVD‐GP ablation that did not perform whole chamber mapping are unlikely to achieve their endpoint, making results difficult to interpret. However, there was no linear relationship between the number of HFS sites tested and the number of AVD‐GPs. There were no identifiable clinical characteristics that predisposed patients to have more AVD‐GP than others.

The AFACT study was a large randomized controlled study that performed adjunctive GP ablation to PVI in AF patients undergoing thoracoscopic AF ablation. There was no benefit in adjunctive GP ablation, but there was an increased complication rate associated with surgical exploration for GP sites. This confirms that the endocardial approach is a safer means for understanding the role of the ANS in AF, but also underlines the importance of understanding the functional pathways triggering AF.^38^, ^39^

## 5 LIMITATIONS

All patients had general anesthesia as HFS causes discomfort in conscious patients. This may have affected the threshold of GP response to HFS. We have studied only the left atrium. The right atrium is also known to have GP as evidenced in previous studies.^2^, ^16^

## 6 CONCLUSION

It is safe and feasible to map the entire LA for AVD‐GP using endocardial HFS before performing a standard circumferential antral PVI procedure. The distribution of AVD‐GPs is highly variable between patients, mandating patient‐specific mapping if the GP sites are to be clinically targeted. Also, we have identified three distinct regions with the highest probability of locating AVD‐GPs that correlates to the findings of previous anatomical human heart studies. Our atlas may be used as a guide for patient‐specific mapping to identify AVD‐GPs more effectively and efficiently. Defining the neural network by whole‐chamber functional mapping may become an important first step in autonomic modification procedures.

## ACKNOWLEDGMENTS

This study was funded by British Heart Foundation, UK (to Dr B C Sandler, Dr N WF Linton, Prof D Francis); British Cardiac Trust, UK (to Dr MY Kim); and Coronary Flow Trust, UK. The group is part of the BHRS Multi‐Centre Trials Group. This work was supported by British Heart Foundation Centre of Research Excellence and Grants, Rosetrees Trust, EPSRC and the NIHR (UK) Biomedical Research Centre.
